# How does regulation influence euthanasia practice in Belgium? A qualitative exploration of involved doctors’ and nurses’ perspectives

**DOI:** 10.1093/medlaw/fwaf003

**Published:** 2025-01-29

**Authors:** Madeleine Archer, Lindy Willmott, Kenneth Chambaere, Luc Deliens, Ben P White

**Affiliations:** Australian Centre for Health Law Research, Faculty of Business and Law, Queensland University of Technology, Brisbane, Australia; Australian Centre for Health Law Research, Faculty of Business and Law, Queensland University of Technology, Brisbane, Australia; End-of-Life Care Research Group, Ghent University and Vrije Universiteit Brussel, Ghent, Brussels, Belgium; Department of Public Health and Primary Care, Ghent University, Ghent, Belgium; End-of-Life Care Research Group, Ghent University and Vrije Universiteit Brussel, Ghent, Brussels, Belgium; Department of Public Health and Primary Care, Ghent University, Ghent, Belgium; Australian Centre for Health Law Research, Faculty of Business and Law, Queensland University of Technology, Brisbane, Australia

**Keywords:** assisted dying, Belgium, euthanasia, health policy, qualitative interviews, regulation

## Abstract

Euthanasia has been legal in Belgium since 2002. Despite extensive research exploring Belgian euthanasia practice, investigations into its governing regulatory framework are limited. Existing studies that consider regulation take a ‘siloed’ approach, generally considering sources of regulation individually, including euthanasia legislation and euthanasia policies. This study obtains insights from providing health professionals on how the Belgian euthanasia regulatory landscape influences their euthanasia practice. We conducted semi-structured, in-depth interviews from September 2022 to March 2024 with eligible physicians and nurses and analysed them using a reflexive approach to thematic analysis. We generated three overarching themes describing the influence of regulation on euthanasia practice: the Act is a valuable, boundary-setting instrument; but the Act is limited, leaving space for gap filling and other forms of regulation; and relying on professional judgment can make practitioners feel vulnerable. Key findings include that practitioners respond to the Act’s non-prescriptiveness and regulatory lacunae by relying on their professional judgment, and that the efficacy of the retrospective euthanasia oversight model depends on physicians’ good faith participation. Policymakers in Belgium and internationally are encouraged to reflect on the implications of Belgium’s euthanasia regulatory model for the consistency, quality, and control of euthanasia practice.

## I. INTRODUCTION

Assisted dying (AD) is being legalized in an increasing number of jurisdictions internationally.^1^ Belgium was among the first to pass its legislation permitting AD, the *Act on Euthanasia* (the Act) in 2002.^2^ Belgium has significant experience regulating euthanasia, and there is extensive research exploring this practice. When the clinical and procedural legal requirements for euthanasia are met, terminally ill and non-terminally ill adults, terminally ill children, and adults who have made an advance request for euthanasia may be permitted to access euthanasia.

For competent adult patients, the law provides that their eligibility must first be assessed by a physician, who assesses whether the patient is experiencing unbearable physical or mental suffering that cannot be alleviated, resulting from a serious and incurable condition. The first physician must refer the patient for a consultation with a second, independent physician, who provides a report on the patient’s unbearable suffering and the incurability of their condition. If the patient is not expected to die in the foreseeable future, there must be a further consultation with another independent physician and a 1-month waiting period from the euthanasia request must be observed. If an adult has made an advance request for euthanasia, this may be activated only if the person subsequently enters an irreversible and permanent state of unconsciousness. Euthanasia for minors under the age of 18 years is very rare, and this process involves further safeguards.^3^ All cases of euthanasia must be declared to the Federal Control and Evaluation Commission on Euthanasia (FCECE). The FCECE reviews all submitted declarations and determines compliance with the law.

It is important that AD is regulated effectively.^4^ Clearly framed, purpose-driven regulation will assist health professionals who are involved in providing AD (‘providers’) to effectively and confidently navigate the AD system and provide high-quality care within the bounds of the legal framework.^5^ In contrast, regulation that is unclear or absent, but needed, could complicate the process for providers, leaving them unprepared to navigate the legal framework and manage their legal obligations, lead to inconsistent or inequitable care provision, or expose providers to criminal or other sanctions for non-compliance.^6^

The legislation described above is just one part of euthanasia regulation in Belgium. Research is increasingly looking more inclusively at euthanasia regulation by considering a wider range of ‘sources’ of regulation than just the law, for instance, euthanasia policies and training programmes on euthanasia.^7^ This wider exploration is important because health professionals have numerous rules and guidelines to follow when navigating and adhering to AD regulatory frameworks. This means that multiple sources of regulation influence their behaviour and decision-making when they are providing euthanasia.^8^ However, more research examining how the regulatory landscape shapes euthanasia practice is needed. This is because existing research has tended to ‘silo’ discussions about regulation by investigating one source of regulation at a time.^9^

Research from a recent scoping review study sought to advance a ‘holistic approach’^10^ to understanding Belgian euthanasia regulation. First, it harnessed the existing literature to comprehensively identify the ‘sources of regulation’ that seek to shape how euthanasia is practised in Belgium, and secondly, the ‘domains’ of euthanasia practice that regulation seeks to govern.^11^ That research identified that there are numerous ‘actors’ who seek to shape Belgian euthanasia practice. These include healthcare institutions and organizations, professional organizations, independent statutory bodies, community organizations, and, to a limited extent, the relevant government ministry. Many of these actors produce regulatory instruments about euthanasia in the form of legislation, policies, standards, advisory documents, training programmes, and system infrastructure.^12^

The second analysis from that review discerned what specific parts or ‘domains’ of euthanasia practice these sources of regulation purport to govern. It found that multiple sources of regulation seek to provide guidance on each identified domain of euthanasia practice, which includes how euthanasia is defined, how to interpret and assess the eligibility criteria, and health professionals’ roles in the euthanasia assessment process.^13^

These studies contributed to and built upon a wider body of literature analysing aspects of Belgian euthanasia regulation, including analyses of the Act and the FCECE and its functions.^14^ Both scoping studies highlighted the complex and multifaceted nature of Belgian euthanasia regulation, as well as the ‘fragmented’^15^ nature of regulation across Belgian care and geographical settings. They identified the need to examine how this regulatory framework influences euthanasia provision in practice, owing to its potential to complicate euthanasia decision-making and cause confusion or frustration for providers as they attempt to discern their obligations. Though this research is useful, we lack insight into how euthanasia regulation works in practice and how it shapes the behaviour and decision-making of the health professionals who provide it. This knowledge is important because it can help us to evaluate and improve the regulatory framework, and consequently, patients’ and providers’ experiences of the euthanasia system. In addition, it may help us to explain the evidence of variation between euthanasia provision across settings^16^ and non-compliance with the legal requirements.^17^ Accordingly, this article seeks to address the research question: how does Belgian euthanasia regulation (including gaps in regulation) influence providers’ euthanasia practice? This study adopts a broad view of regulation and defines it as:

the sustained and focused attempt to alter the behaviour of others according to defined standards or purposes with the intention of producing a broadly identified outcome or outcomes, which may involve mechanisms of standard-setting, information-gathering and behaviour modification.^18^

## II. METHODOLOGY

This study is part of a wider investigation of euthanasia regulation in three jurisdictions: Belgium, Canada, and Australia.^19^ We used qualitative, in-depth semi-structured interview research design. We adopted a critical realist position in this research.^20^

### A. Eligibility to participate

Doctors and nurses were eligible to participate if they spoke English or Dutch and had been involved in the euthanasia assessment of at least two patients in the past year. The intention of this latter requirement was to include practitioners with both more and less experience in providing euthanasia. We included both doctors and nurses in the study. Nurses cannot undertake eligibility assessments or administer the life-ending medication. However, nurses often have a role in the euthanasia decision-making process (such as by exchanging information with the physician about the person’s wishes and condition), provide ongoing care to the patient and their loved ones, and provide support to physicians, including with the administration of the life-ending medication.^21^

### B. Participant recruitment

We recruited participants through the professional networks of the research centre led by the Belgian authors (K.C. and L.D.), advertisement by relevant organizations, and using a snowball approach. We purposively selected participants to seek diversity (heterogeneity) with respect to participants’ profession; level of experience; religious, cultural, and ethnic background; and gender identity. Participants’ consent to participate was obtained prior to the interview.

### C. Data generation

We developed an interview guide (Supplementary Material 1) in which the prompts (not the main questions) were iteratively adapted before each interview to reflect learnings from previous interviews and the participant’s health profession and work setting. The interview guide stepped participants through the euthanasia process from receiving a request for euthanasia through to administration of the life-ending medication and aftercare. At each stage, participants were asked about what ‘sources’ of regulation they rely on, if any, to help them navigate that part of the process, such as legislation, policies, professional standards, training programmes, advisory documents, and system design.^22^ Participants were also prompted to think generally about sources of euthanasia regulation, interactions between sources, and how they influence their euthanasia practice.

We conducted interviews using Microsoft Teams videoconferencing between September 2022 and March 2024. We conducted the interviews in English (led by M.A., with L.W. assisting in several interviews), Dutch (led by K.C.), or in English with a Dutch-speaking member of the research team present to assist (M.A. leading, K.C. or L.D. assisting), according to the participant’s stated preference. All interviews were recorded and transcribed verbatim by a professional transcription service. We gave all participants the opportunity to review their transcripts and add or remove information.^23^ Dutch transcripts were then translated into English by a translator with experience conducting research on euthanasia in European jurisdictions. Some participant quotations provided below have been adapted to reflect the participant’s meaning in English, where necessary, though their meaning has not been changed. In addition, some information in these quotations has been de-identified where necessary to protect participant confidentiality.

### D. Data analysis

M.A. used a reflexive approach to thematic analysis to analyse the data.^24^ This approach facilitated the use of regulatory theory to inform the study design. In addition, this approach facilitated the use of an inductive approach to coding to generate both semantic (surface-level) and latent (underlying) themes to address the study’s exploratory research question.^25^ This approach involved note-taking when the transcripts were first read to ensure data familiarization. Next, M.A. coded all interviews twice resulting in initial codes. The first 10 interviews were coded first, simultaneously with further data generation, culminating in a preliminary coding framework that facilitated the same process to be applied to the remaining transcripts as data generation progressed. Initial themes were generated as codes were altered, reorganized, and arranged. Themes were merged, adapted, and settled as a result of discussions within the research team.

No further interviews were undertaken when all authors determined that sufficient ‘information power’ for the study had been reached.^26^ This determination was made by the research team, informed by the study’s specific research objective, the inclusion of participants both with considerable experience providing euthanasia and little experience providing (reflecting the purposive approach to participant selection), and preliminary analysis.

### E. Reflexive research practice

M.A. maintained a reflexive journal throughout the coding and theme-generation processes.^27^ Reflections on the role of the research design, as well as researchers’ characteristics and professional backgrounds on the data were included in the reflexive journal. Reflexive notes were also made after each interview. In particular, M.A.’s entries probed the influence of her position as being geographically, culturally, linguistically, and professionally separate from the research participants, as well as the effectiveness of measures implemented to bridge those differences (where appropriate to do so) and establish rapport. The entries in the reflexive journal were used to inform subsequent coding and theme generation.

### F. Reporting of the study

This study is reported in accordance with the consolidated criteria for reporting qualitative studies (COREQ) (a completed checklist is presented in Supplementary Material 2).^28^ This study is also reported (and was conducted, insofar as appropriate and applicable) in accordance with the Reflexive Thematic Analysis Reporting Guidelines (RTARG)^29^ (Supplementary Material 3 presents these guidelines and indicates how these recommendations are reflected in the conduct and reporting of this article).

### G. Ethical approval

The study was reviewed and approved by the Medical Ethics Committee of the Brussels University Hospital with reference BUN 1432022000043 and by the Queensland University of Technology Human Research Ethics Committee with reference 2000000270.

## III. RESULTS

### A. Participant characteristics

A total of 20 interviews were conducted (see Table 1 for a summary of participants’ characteristics). The median interview length was 94 min (range: 65 min to 111 min). Though participants were selected purposively to ensure diverse cultural and ethnic identities, all participants identified as having Dutch-speaking Belgian ethnicity. Most participants spoke English in the interview (*n* = 14), while others were supported to participate in English (*n* = 4) or participated in Dutch (*n* = 2).

**Table 1.** Participant characteristics.

Participant characteristic	No. of participants (*N* = 20)
Gender identity	
Female	11
Male	9
Age category (years)	
<30	1
31–40	2
41–50	7
51–60	5
61–70	2
>70	3
Health profession	
Physician—general practice specialty	5
Physician—medical specialty	9
Nurse	6
Location of primary practice	
City	11
Town	7
Rural municipality	2
Region of practice	
Flanders	19
Brussels	1
Main setting of practice	
Community	7
Hospital	11^a^
End-of-life consultation centre	2
Years of experience	
1–10	4^b^
11–20	4
21–30	4
31–40	4
41–50	4
Number of cases of euthanasia involved in	
<10	1
10–19	2
20–29	2
30–49	3
50–99	4
100–500	3
>500	5

a One participant who currently works in the hospital setting also has significant (though previous) experience working in the community setting.

b One participant indicated that they had worked in their current role for 9 years and it was not clear if their work history extended past this role. The participant was not able to be contacted to clarify.

### B. Themes describing the influence of regulation

**Figure 1.**
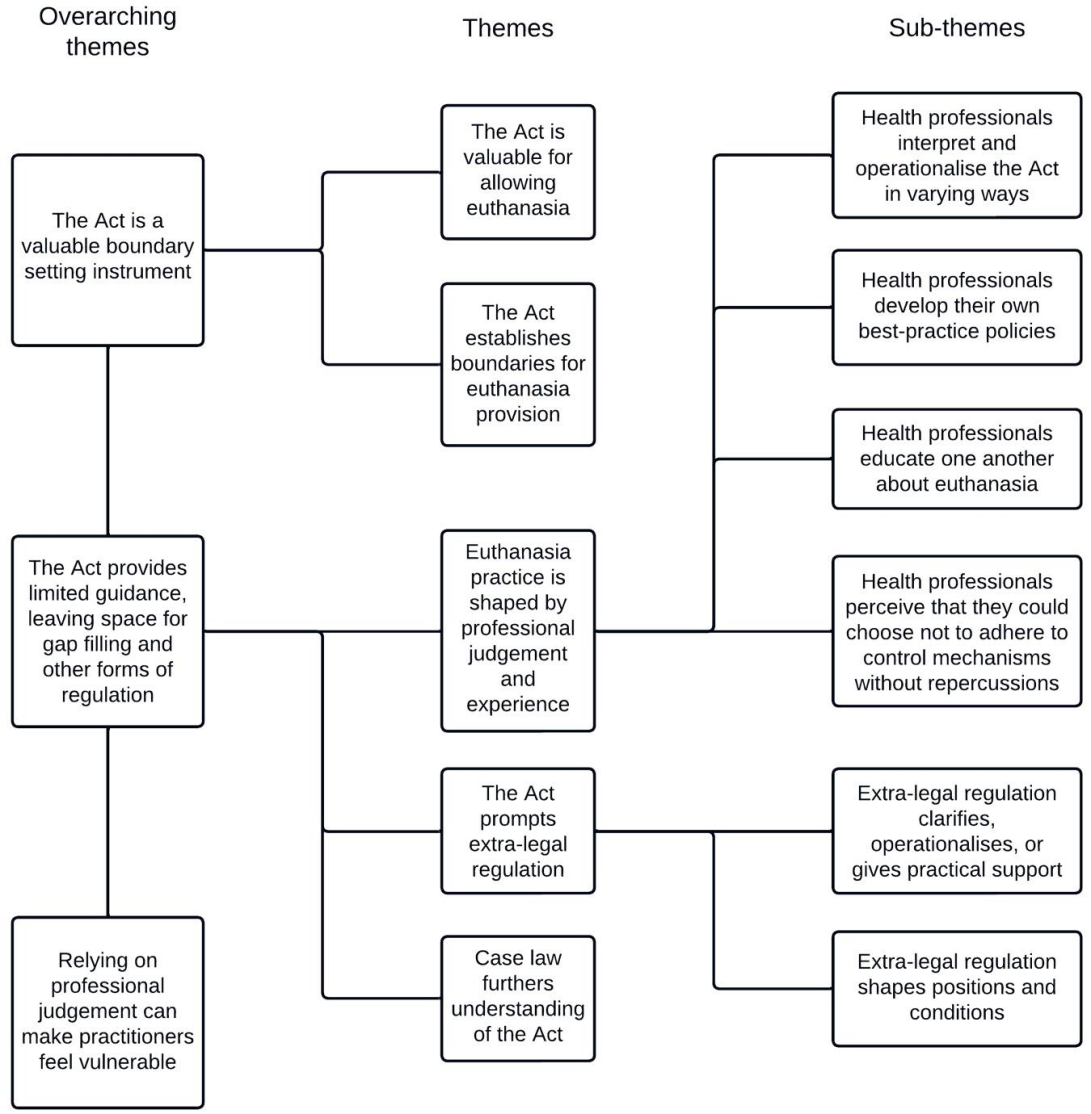
Schematic diagram reflecting the overarching themes, themes, and sub-themes and the relationships between them.

We generated three overarching themes, comprising five themes and six sub-themes. We developed a schematic diagram to reflect the relationships between the themes (Fig. 1).

#### 1. Overarching Theme 1: the Act is a valuable, boundary-setting instrument

This theme describes participants’ perceptions that the Act is an important instrument both for permitting euthanasia and establishing the boundaries for euthanasia practice.

##### a. Theme 1a: The Act is valuable for allowing euthanasia

Most participants expressed appreciation that the Act permits them to provide euthanasia as an end-of-life option. Many described the secrecy that surrounded euthanasia prior to the Act’s introduction and identified that its legalization brought conversations about death and dying into the open, doing much to stimulate broader discussions with patients about their end-of-life care.

One participant expressly referred to the importance of the Act for permitting euthanasia for patients with mental disorder. While they observed that euthanasia was performed prior to the enactment of the law (albeit in secret as it was illegal), this was only in acute situations where the patient was terminally ill. Accordingly, the Act has been essential for providing access to euthanasia for patients with mental disorder.

One nurse participant cited the Act’s permissive and protective functions when describing their institution’s euthanasia policy, which was based closely on the law as ‘something we can point to and use as a shield, so to speak’.

##### b. Theme 1b: The Act establishes boundaries for euthanasia provision

Some participants referred to the Act as establishing necessary boundaries for euthanasia practice that were not in place prior to 2002 when euthanasia was unregulated (and illegal). One participant remembered their colleague saying ‘now it will be more difficult to perform euthanasia’ when the Act was introduced because it established boundaries that were not previously in place. Another participant identified that the legal procedure established in the Act was necessary to prevent adverse events and inappropriate care provision.

…*before the euthanasia law, the fact that there wasn’t a legal procedure and no one really knew how to do it, there was a lot of messing around by physicians, by family members, in some cases nurses, who tried to help the patient by giving an overdose of morphine…. Or we were sometimes called to emergency when a physician had tried to ‘help’ a patient and the patient didn’t die.* Participant 22, nurse

Many participants identified that the Act gives them a clear understanding of who can ask for euthanasia, and what is not permitted. Some expressly referred to the Act as being the ‘basis’ and the ‘starting point’ for euthanasia, as ‘foundational’ for understanding how to provide euthanasia and something that they would always ‘stick to’.

One participant expressed that the boundaries introduced in the Act were important at the beginning of the legal framework, but no longer necessary as euthanasia has become a normal part of medical practice. They reported considering that there may not be legislation regulating euthanasia in Belgium in the future.

#### 2. Overarching Theme 2: The Act provides limited guidance, leaving space for gap filling, and other forms of regulation

Participants explicitly and implicitly acknowledged that the Act, though important for authorizing and confining the scope of euthanasia (see Overarching Theme 1), does not and cannot offer comprehensive guidance on providing euthanasia. Participants described the framing of the Act as ‘vague’, ‘subjective’, ‘murky’, ‘fluid’, as giving them ‘freedom’ to provide, ‘room to fill in’, and ‘a lot of space in our patient-physician relationship’.

While some participants considered the law’s subjectivity appropriate in some cases, in others, they wished for more clarity. Some participants expressed that making the Act more specific might make it more difficult to apply. Participants identified two parts of the Act that they did not believe could be more closely defined. These were the eligibility requirements of ‘unbearable suffering’ and the provision stating that a patient does not have to include their family members in the euthanasia process. Regarding the latter, almost all participants identified that best practice is to include the patient’s family, but they uniformly agreed that the Act could not mandate that family members should be involved, due to the complexities of familial relationships.

In contrast, some participants readily identified certain situations in which the Act could and should clarify practice. These included specifying what is meant by the independence required of the consulted doctors in the assessment process; clarifying how to assess the eligibility of patients with mental disorder; and reconfiguring the assessment process to reflect decision-making within multidisciplinary teams. The tension between the Act needing to be more prescriptive whilst remaining flexible was expressed by this participant:

*Well, in some areas the law clearly could be more specific, I think. If you think about the independence of the second doctor, well, it would be better for it to have a clearer definition of ‘independent’ … what we’ve got to realise is that the same law is used in very different settings: in hospital setting, in home care, in nursing homes. So it should be appropriate for all these settings. In some areas, probably it’s not useful for the law to be too specific….* (Participant 5, general practitioner)

##### a. Theme 2a: Euthanasia practice is shaped by professional judgment and experience

As a result of the Act’s broad framing, doctors, nurses, and healthcare teams shape euthanasia practice according to their own professional judgment and experience. This sub-theme encompasses four sub-themes.

###### i. Sub-theme 2a(i): Health professionals interpret and operationalize the Act in varying ways

Participants reported that they interpret and operationalize broadly framed provisions of the Act. Several legislative provisions were described as being ‘vague’, ‘murky’, ‘never black and white’ including the requirements that the patient’s eligible condition must be incurable and causing unbearable suffering. Another example is the legislative requirement to ‘consult’ the patient’s nursing team if applicable; one participant expressly identified that ‘consult’ could be operationalized in several ways ranging from a brief mention of the request to the nursing team to a considered discussion and deliberation.

Two legislative provisions were frequently identified by participants as being open to being interpreted and operationalized in different ways. First, the Act establishes different assessment pathways for patients based on whether they are expected to die within the foreseeable future. Some participants reported applying a specific time limit to facilitate this assessment. Using a 3-month life expectancy was most commonly reported by participants working in palliative care and oncology, and some others reported using a 6 or 12-month period. Other participants reported using non-temporal operationalizations, including the number of hospitalizations the patient had undergone in a recent period, adapting an existing tool such as the ‘SPICT scale’, which facilitates a determination of formal palliative status,^30^ or using the patient’s diagnosis to determine their life expectancy. Regarding the latter, for example, one participant explained that patients with Parkinson’s disease are rarely considered terminal, whereas patients with motor neurone disease are always considered terminal.

Secondly, several participants also reported variability in how the term ‘independent’ with respect to the consulted physician or physicians, a term not defined in the legislation, is applied in practice. Some considered it to require that there is no therapeutic relationship between the patient and the second physician, but that there could be some relationship between the first and subsequent doctors. Many participants identified several possible interpretations for this term.

*Independence is something that you yourself have to feel yourself. So the law is not saying it has to be this or that.* (Participant 6, general practitioner)

###### ii. Subtheme 2a(ii): Health professionals develop their own best-practice policies

All participants explicitly or implicitly identified that they develop and apply rules or ‘policies’ for their own clinical best-practice euthanasia provision. These rules are based on learnings from experience and relate to the whole euthanasia process from discussing euthanasia with patients through to aftercare. Examples of policies developed and applied by participants and the area of euthanasia practice they relate to are set out in Table 2.

**Table 2.** Examples of best practice rules health professionals develop and apply to support their euthanasia practice.

Area of euthanasia practice	Examples of best practice measures participants employ
Discussing euthanasia and exploring the patient’s request	Raise euthanasia in the context of a general end-of-life discussion, or alternatively, always wait for the patient to raise euthanasia. The first discussion that a health professional has with a patient about euthanasia should be face-to-face, without the patient’s family members present. Specific language should be used by the health professionals exploring the patient’s request, in particular, to determine whether the patient wishes to die, or to enter a permanent state of sleep or unconsciousness. Key to exploring the patient’s request for euthanasia is discerning what is motivating their request and identifying whether other measures, for example, increasing pain medication can resolve the request. A discussion about euthanasia with the patient should explore the patient’s expectations and concerns. Educate patients that euthanasia may not be the best solution in certain situations (that a good death is the goal, not euthanasia itself). The situations in which a patient’s advance request can be acted upon (and not acted upon) should be clarified with the patient and their loved ones as early as possible, as this is a common source of confusion and/or conflict.
Who is involved in decision-making/the euthanasia assessment trajectory	Ideally, the patient’s family members should be involved in the patient’s euthanasia assessment trajectory to maximize a positive experience for all (patient, providers, and family) unless exceptional circumstances apply. It is important that the attending physician has a longstanding relationship of trust with the patient and understands the patient’s request (rather than providing euthanasia on demand to new patients). Where possible, nurses should inform the euthanasia decision-making process, and decisions should be a team decision (including psychologists and other health professionals). Nurses should assist patients to clearly articulate their request for euthanasia (or other end-of-life option) and express it to their treating physician. In the hospital setting, the patient’s general practitioner should be contacted to ascertain whether the patient has discussed euthanasia previously, their family’s response, etc.
The form and content of the euthanasia assessment process	An extra assessment should be undertaken when the independent consultant does not find the patient’s suffering to be constant, unbearable, incurable, or both. Euthanasia should not be seen as an urgent treatment option. Practitioners should take the time they require to carefully consider the patient’s eligibility and whether they are comfortable providing euthanasia for the patient. Euthanasia should not be provided in a rush, or in emergency situations where the practitioner does not feel as though they have had time to properly consider the request. For patients whose request is based solely on mental disorder, specific, additional measures should be part of the assessment trajectory, including the adoption of a two-track approach and the observation of a 6-month waiting period. A long period may be needed to accurately and comprehensively assess a request based solely on mental disorder. In this time, it is important to engage with the patient’s family, if they are amenable to that. In the euthanasia assessment process, it is important for health professionals to place the patient at the centre and to listen to the patient, as it can be easy to become preoccupied with legal and medical issues, and evidence about their medical condition, rather than the patient’s individual experience.
Documentation	Each time a patient raises euthanasia with a health professional, this should be documented in their medical records, and proof of conversations should also be documented (this can help to establish the ‘repeated’ nature of the patient’s request).
The technical performance of euthanasia	Euthanasia should never be performed by a single physician alone. The patient’s loved ones who are present when euthanasia is being provided should be educated about the patient’s possible response to the administration of the life-ending medication. Intravenous administration of the life-ending medication is to be preferred over oral administration, especially where the patient’s ability to drink all the required liquid is not clear. The substance does not taste pleasant which can be a difficult experience for the patient and their loved ones.
Provider aftercare	Health professionals should ensure there is a break between performing euthanasia and continuing with their daily work.

###### iii. Sub-theme 2a(iii): Health professionals educate one another about euthanasia

Most participants reported that they learned about euthanasia and how to provide it through observation, mentorship, and experience. Many participants completed their undergraduate training prior to the commencement of the Act, meaning that they received very little or no formal training on euthanasia. Some, mostly younger participants have undertaken specific training on euthanasia and end-of-life care; however, they identified that they and other providers must be self-motivated to undertake this training as it is not mandatory.

*But we do not have any education on euthanasia or even palliative care in our medical education, we don’t have it on a standard basis. So not every physician that graduates here in Belgium has education in euthanasia or palliative care. So… we need to look it up and we need to do it ourselves.* (Participant 15, medical specialist)

Participants who are highly experienced in providing euthanasia or who have completed the specific training play a key educative role in supporting less experienced colleagues in their work settings. These more experienced providers often become known as educators, and sometimes they have developed their own documents about providing euthanasia, which are used by others as educational tools. Such providers include members of palliative care teams in hospitals and experienced nurses in the community setting, and they are known for having expertise and for providing hands-on support in euthanasia cases. One general practitioner with an educative role reported developing a document, or a ‘euthanasia roadmap’, which they give to less-experienced practitioners. When questions arise in practice and support is needed, colleagues are the most common source of guidance sought.

###### iv. Sub-theme 2a(iv): Health professionals perceive that they could choose not to adhere to control mechanisms without repercussions

Almost all participants identified that the nature of the legislative control mechanisms is such that providers can effectively use their professional judgment to decide whether and how they comply with them. This is because, they identified, they would either be very unlikely to face repercussions if they failed to comply, or because the Act’s framing means that these control mechanisms can legally be avoided. Despite the regulatory system being unable to compel practitioners’ compliance with these obligations, all participants reported that they adhere to the legislative control and oversight mechanisms and participate in good faith.

Three control mechanisms were discussed in this context: retrospective reporting of euthanasia to the FCECE; the internal control provided through independent consultations with another physician; and the obligation on physicians who conscientiously object to euthanasia to refer the patient to another physician.

First, many participants reported that they perceive the Act’s retrospective oversight mechanisms to be ineffective, insofar as they permit physicians to decide both *whether* and *how* they report euthanasia. Regarding the former, most participants considered that if they did not report euthanasia, this omission would not be detected by the FCECE. One participant reported forgetting to report euthanasia because immediately afterwards they went on leave. Another participant described acting as the independent consultant in two cases, neither of which were reported to the FCECE. The participants knew this because they asked each attending physician, subsequent to performing euthanasia, whether the case was reported. In the first case, the attending physician said that they forgot to report it, and in the second, the attending physician stated that they never report euthanasia because they know they have performed it correctly.

In terms of *how* they report to the FCECE, participants described that by relying on self-report, the accuracy of the information relies on physicians’ honesty and fulsomeness, meaning that it could be easily falsified or have non-compliant details omitted. One participant stated that reporting is ‘an evaluation of how doctors are able to fill a document in, not what they have really done’. Participants who had made an error in their report to the FCECE were provided with an educative letter and no sanction: one forgot to observe the 1-month waiting period for a non-terminally ill patient, and the other made an administrative error on the report. See Table 3 for an overview of the reasons participants considered the FCECE’s ability to control euthanasia practice to be limited, along with participant quotations.

**Table 3.** Reasons cited by participants that the Act’s retrospective oversight mechanisms are ineffective, making space for them to exercise professional judgment with respect to compliance, with illustrative participant quotes.

Reason	Illustrative quote(s)
The retrospective oversight mechanisms cannot identify non-reported cases nor falsified information	[Many] *years ago… for one of my own patients and then after two weeks, I was like, ‘I didn't report.’ And there is not—they* [the FCECE] *cannot control it. That's one of the gaps in the legislation…. But then you know that it's quite unrealistic to make the report because it was someone who was dying, the procedure was OK*, [I] *did everything, that was OK… I can imagine that a lot of reports are not made because they* [physicians] *will say, ‘It was OK, I did everything* [correctly]*,’ and it happened to me too.* [Participant identification number withheld to protect confidentiality] *One issue of this reporting is that the full control of this report is in the hands of the physician performing the euthanasia. So the independent assessment reports are not included. So I take out of the independent assessment reports as much as I like. So you can imagine if an independent assessment report says I confirm this patient requests euthanasia. He suffers from advanced bowel cancer but at present he’s confused. He’s severely depressed. His family members say he is making plans for the future. His oncologist has just started treatment, we are first trying chemotherapy, there are several other lines as well and so on. What I can perfectly do is copy-paste the first sentence and omit all the rest. That’s possible. No one would ever know.* (Participant 4, medical specialist)
The FCECE’s significant workload means that not all cases can be checked	*I think what doesn’t work—so that, you could say I still have a great faith in the majority of doctors. I think there will always be a minority who do very strange things. What doesn’t work is the control system. They actually don’t have time to do it decently.* (Participant 19, medical specialist) *There are a lot of euthanasia files each year so not all of them are checked. The committee does not work full time.* (Participant 13, nurse)
The FCECE does not have access to the information it needs to exercise substantive oversight, so is hamstrung by its legislative role	*… because of course you can write in that document everything you want. You also need to fill in the short description of the advice of the independent physician, but it’s not checked with what the independent physician actually wrote. So you can actually write down whatever you want. Or even say that there was an independent physician but there wasn’t one. It’s actually possible, because as far as I know it’s not checked. Also, when I was the independent physician, I had never been called by the Commission to ask, ‘Do you know this case, and have you been there, this independent physician?’ I have never had that request or that question. So everything that is on the document is everything you need, actually, for the euthanasia. But I don’t think they really check the validity of everything that is written on that document. Because they have no access to the medical file*. (Participant 15, medical specialist)

In contrast to the above discussion, two participants described situations in which the retrospective oversight mechanisms were experienced as effective, even punitive. One participant was ‘profusely sweating’ when they received a letter from the FCECE in respect of a case of providing euthanasia. A nurse participant described that a colleague physician no longer provides euthanasia after having been ‘frightened’ by receiving a letter from the FCECE for forgetting to include a required document.

Secondly, most participants identified that it is problematic that, under the Act, the advice given by the independent consultant or consultants can be ignored by the attending physician. They expressed that these second and third opinions do not act as the control mechanism that they were intended to be, and that they have little value because they are not binding. One participant reported that all cases in which they were the independent consultant resulted in the patient accessing euthanasia, although some of their reports had stated that, in their view, the patient’s suffering was not unbearable or their condition was not incurable.

Thirdly, some participants reported that the legal duty for physicians with a conscientious objection to refer the patient to another physician or relevant service is not always respected. One participant considers that this obligation on conscientious objectors needs to be strengthened, considering how easily it can be circumvented, having observed colleague physicians not adhering to this obligation. Some nurse participants expressed that they have a key role in advocating for patients when they have expressed a wish for euthanasia (part of a larger role in advocating for the patient’s wishes for their end-of-life care in general). This means clearly explaining to patients how they should articulate their wish for euthanasia to the treating physician so that there can be no doubt that the patient is asking for euthanasia. Some participants identified that some instances of non-adherence to this requirement may be unintentional, owing to a lack of knowledge.

*This week we were at the ward with a patient who asked a very clear euthanasia question, and he has been for three weeks. And the supervisor there says, ‘I’m not performing euthanasia out of principle, this is not the type of ward to do that on, and this is not the type of pathology to do that on.’ And I’m like, ‘OK, and how are you going to fix this?’ And he was like, ‘What do you mean, how am I going to fix this?’ I’m like, ‘Yes, you have the obligation to refer your patient.’ ‘Really? Do I?’ People don’t know.* (Participant 12, medical specialist)

##### b. Theme 2b: The Act prompts extra-legal regulation

Participants identified that sources of regulation other than legislation have been produced or adapted to guide euthanasia practice. Participants described these sources as having two main influences in practice: clarifying, operationalizing, or giving practical support to providers; and shaping positions and conditions. These sub-themes are described below.

###### i. Sub-theme 2b(i): Extra-legal regulation clarifies, operationalizes, or gives practical support

Participants referred to several sources of regulation which they consider to clarify, operationalize, or give support to euthanasia provision. Four of these appeared to be particularly influential in shaping euthanasia practice: training programmes offered by Palliative Care Flanders and Life End Information Forum (LEIF), institutional and health network policies, LEIF advice and resources, and palliative care teams and written palliative care guidelines. Palliative Care Flanders is an umbrella organization supporting those providing palliative care services. LEIF is an end-of-life consultation centre that provides training on euthanasia and end-of-life care and facilitates referrals to independent second physicians as required by the law.^31^

Table 4, below, provides an overview of participants’ perceptions of each of the four sources of regulation they identified as facilitating or supporting their euthanasia practice.

**Table 4.** Summary of participants’ insights on how influential sources of regulation facilitate or support euthanasia practice, with illustrative participant quotations.

Influential source of regulation	Useful, facilitative, or supportive functions of that source of regulation	Illustrative participant quotation
Training programmes developed by Palliative Care Flanders and Life End Information Forum	Clarifying their legal obligations	[The LEIF training is] *very helpful. If I didn’t get the training, it would be very difficult* [to provide euthanasia]. *Because I’m also young, I don’t have experience in another department in the hospital or something like that. I just came from school to the hospital. So, it was very helpful. Really good training.* (Participant 16, nurse)
	Explaining the legal process	
	Supporting the completion of required paperwork	
	Providing specific medication protocols	
	Offering ongoing case discussions with peers, ‘intervisions’^a^ and peer support	
Institutional and health network policies	Containing practical, step-by-step guidance, such as flow-chart or checklist-type, which are useful to follow in practice	*Normally, the legislation says that the patient can autonomously receive euthanasia, without their family knowing. In our hospital, that’s just not done. We get those requests from patients sometimes. It’ll be maybe once a year that we get a request like that from a patient like, ‘Can you give me the injection without my wife and children knowing? I don’t want to burden them with it.’ Do we do that? No, then we don’t go ahead with that euthanasia. That might seem a bit like we’re playing God, but it’s not about that. It’s about the fact that we realise what kind of pressure that places on the shoulders of someone who hears after the fact that the husband or wife has died. You have to provide an explanation for how they died.* (Participant 10, nurse (translated))
	*Institutional policies*	
	Containing specific guidance on the euthanasia process in the institution such as: defining when the terminal assessment trajectory should be applied, whether and how euthanasia should be discussed, who should be involved in the assessment process, what their role should be, how to involve patients’ family members in the process, how to understand certain eligibility criteria, protocols for prescription, administration, and substance disposal, aftercare, and debrief.	
Life End Information Forum resources and advice including the *Leifdraad*	Contains guidance on euthanasia for practitioners working in the community setting, who unlike their colleagues working in institutions, lack a central policy to refer to.	[Referring to the LEIDdraad] *It’s like the Bible we use, that’s where you get your info. It’s only 18 pages, but it’s very clearly written, it has extremely detailed accounts of injecting, of which products you can absolutely not mix because they clog the lines. And I think the most info is in four pages, like if you have those four pages, you’re fine.* (Participant 3, general practitioner)
	*Leifdraad*	
	Containing checklists	
	*Advice function*	
	Offering ability to discuss cases, seek information, or to check a person’s eligibility for euthanasia, find an independent physician, and receive hands-on support at the administration of euthanasia.	
Palliative care professionals and written palliative care guidelines	Influencing practice in both the general practice and institutional settings.	*The law is really on the individual choice of a conscious patient. And that’s a good thing. But in palliative care we work for a system. We work with families, with proxies, and also in the case of euthanasia, it’s important to have the same attention and the same work with families, with proxies. And there is nothing about that in the law. And the law is, on several items, is quite vague, in fact. So developing a good view on these vague issues, but also bringing in, I think, quality standards of palliative care also in a trajectory of euthanasia is quite important.* (Participant 5, general practitioner)
	*Palliative care professionals*	
	Housing euthanasia expertise in the hospital context	
	Producing specific guidelines on euthanasia for their colleagues	
	Involved in the process for all patients who request euthanasia (in accordance with the hospital policy)	
	*Pallialine guideline*	
	Informing individual practitioners’ decision-making	
	Forming the basis for several institutional policies on euthanasia	
	Describing optimal euthanasia care	
	Describing how shared-decision-making processes used in palliative care can be used effectively in euthanasia-decision making	

a An intervision is a group meeting in which attendees can discuss real clinical cases with colleagues and problems experienced in practice.

###### ii. Sub-theme 2b(ii): Extra-legal regulation shapes positions and conditions

Several participants working in institutions reported that their practice is shaped by their institution’s stance towards euthanasia. Physicians and institutions are at liberty to add extra conditions to the legal process to narrow the circumstances in which euthanasia can be provided. This constrains employees from providing euthanasia only in the circumstances and according to the processes permitted by their institution (as while these conditions may not be required by the legislation, it may be difficult for physicians to deviate from them).

Some participants considered these added requirements to be useful or important. These included that some institutions mandate a longer waiting period for individuals whose request is motivated by mental disorder, that consultation must occur with a palliative care support team before the patient’s request can be advanced, and that an advance request for euthanasia cannot be applied in the period of unconsciousness immediately preceding death.

Many participants described that more restrictive policies towards euthanasia were generally held by institutions with Catholic foundations. However, they equally reported that many institutional stances that were highly restrictive towards euthanasia originally have transitioned to a less restrictive position. One participant observed this transition in their own institution, which now adopts a policy that is deferential to providers (rather than one that is actively supportive of euthanasia). Despite this, the participant identified that physicians working in the institution can still be reluctant to provide euthanasia owing to its origins.

*The* [institution] *has no input… well they are not involved and they have no voice in the decision of a physician…. But the physicians are always reluctant because they don’t know what* [the institution’s management] *will have to say about it. Given the fact that I’m working in a hospital which has a… Catholic tradition.* (Participant 22, nurse)

Several participants reported needing to correct patients’ assumptions that they will not be able to access euthanasia in institutions that are Catholic or have historically been Catholic.

Many participants reported that there is scope for professional associations (for both medicine and nursing) to be instrumental in shaping their euthanasia practice, as this has not previously been the case. One nurse participant identified that their nursing association adopts a risk-averse stance towards euthanasia, which can prevent some nurses from becoming involved in providing euthanasia.

*They* [nursing associations] *are really not stimulating nurses to get involved. On the contrary, ‘Stay out because it’s a minefield. You’d better stay out, it’s clearly something between physicians and patients.’ So I think it’s only the palliative care nurses who want to remain involved.* Participant 22, nurse

Guidelines produced by the Flemish Association for Psychiatry (VVP) and the Order of Physicians were reported to be influential and useful in the context of patients whose request is based on mental disorder. These guidelines recommend applying particular operationalizations of the legal requirements and adding conditions when assessing these patients for euthanasia. Specifically, some participants highlighted their utility in demonstrating the need for greater care (to protect both the patient and physician) when assessing these patients. One participant expressly stated that they apply some of the protocols in the VVP guideline.

##### c. Theme 2c: Case law furthers understanding of the Act

Several participants reported that case law has supplemented their understanding of the legislation.

Most participants referred to the high-profile Belgian euthanasia criminal trial decided in 2020.^32^ The three doctors involved in the euthanasia assessment of a person whose request was based on a mental disorder were prosecuted and ultimately acquitted. Many participants reported that the trial had caused them to reflect on their interpretations of the legal requirements, such as the meaning of the term ‘independent’.

*They say: ‘look, if you treat a patient as a specialist for one or two times, then you're still independent. But if you're on oncologist, and followed your patient for two, three, four, five years, you're not independent to that patient.’ That's what they say.* (Participant 6, general practitioner)

Three participants referred to a case in 2015 in which the FCECE forwarded a euthanasia case to the public prosecutor. The situation, which was filmed and featured in an Australian documentary,^33^ concerned the euthanasia of an 85-year-old woman whose physician handed her the life-ending medication, which she then drank. The physician was not ultimately prosecuted, and two participants cited this matter to demonstrate that physician-assisted suicide is not considered euthanasia under the Act.

#### 3. Overarching Theme 3: Relying on professional judgment can make practitioners feel vulnerable 

Several participants referred to the significant influence that the criminal trial, referred to above, has had on their approach to euthanasia assessments. For many, this case signalled that euthanasia practice is now vulnerable to external (ie legal) scrutiny in a way that it was not previously, and it has caused some participants to be fearful of providing euthanasia in some or all contexts. Most participants expressed one of the following two views in relation to the trial. Some reported that the trial has made their euthanasia decision-making more thorough. These participants identified that the Act’s subjective framing, which allows for a breadth of approaches when conducting euthanasia assessments, requires them to be more cautious in undertaking these assessments in the future after the trial. One participant stated that they would now refer a patient for further consultations where the independent consultant considered that the patient was not eligible.

*So five years ago we would perform. Now I would look for a third advice. I would look until I found somebody who says, ‘Yes, of course.’ And the more ‘no’s’ I would get back, I would clearly start to question my own judgement then*. (Participant 3, general practitioner)

Other participants, though affected by the trial, reported that their euthanasia decision-making was already sound, so their processes did not need to be made more thorough to ensure they were legally protected when providing.

## IV. DISCUSSION

### A. Summary of themes

This study is the first to investigate the influence that the euthanasia regulatory framework has on the practice of health professionals who provide euthanasia in Belgium. We generated three overarching themes in the analysis: the Act as a valuable, boundary-setting instrument; the Act provides limited guidance, leaving space for gap filling and other forms of regulation; and relying on professional judgment can make practitioners feel vulnerable. We generated five themes and six sub-themes that correspond to these overarching themes (see Fig. 1).

### B. Interpretation of themes

The themes demonstrate that the Act is a valuable part of the Belgian euthanasia regulatory framework. Participants identified that they appreciated the Act’s permissive quality and acknowledged the regulatory benefit of having a law regulating euthanasia.

Participants identified that the Act is not the only source of regulation which shapes their practice, and they identified others, including case law, the FCECE (and more broadly, the system’s retrospective oversight mechanisms), training programmes, policies, healthcare teams, and professional standards. Often, these sources were reported by participants as facilitating or supporting them in their euthanasia practice. In other cases, they identified sources of regulation as being ineffective, confusing, or as making them feel vulnerable.

The themes also show that providers respond to the Act’s broad framing and regulatory lacunae by relying on their professional judgment and experience. In fact, professional judgment and experience appeared to be more important for participants than all sources of regulation other than the law. A particularly important finding is that participants in the study reported that they comply with the obligation to report euthanasia to the FCECE, despite the retrospective model of oversight being unable to compel practitioners’ compliance with this obligation. In other words, the regulatory framework is supported by physicians’ good faith participation. Notwithstanding reports of infrequent non-compliance with this obligation, and the possibility of non-compliance, physicians reported that they generally adhere to the requirement to submit to oversight. Accordingly, this model of oversight reflects a firm reliance on the professional and deontological integrity of physicians, and physicians comply owing to their professional judgment (rather than just based on their knowledge of legal consequences).

The themes present an opportunity to consider how AD regulatory frameworks evolve in advanced AD regulatory settings. Many participants in the study reported developing their own best-practice euthanasia policy of engaging in shared decision-making for euthanasia involving multidisciplinary teams, including nurses and psychologists. The Act does not reflect that approach and instead reflects decision-making within the confines of the physician–patient relationship.^34^ This disconnect between law and practice may be indicative of maturation in dominant approaches to clinical decision-making and reflect a shift in medical culture.

In a similar vein, one participant reported that they do not think that there will be a law on euthanasia in Belgium in the future (theme 1b). They reported that, while it was important at the beginning to permit a previously illegal end-of-life option, euthanasia has become embedded in regular medical practice and will, in the future, be regulated like other end-of-life options are, that is, through clinical guidelines and not legislation. Though this view was not expressed by other participants, this too may demonstrate how AD regulatory systems might mature or evolve over time.

### C. Contribution to the existing literature

This research adds to the existing literature on Belgian euthanasia practice. It provides empirical evidence demonstrating the array of sources of regulation which actively seek to shape euthanasia practice^35^ and reports health professionals’ views on how they do so. Previously, only one study had sought to provide a comprehensive list of these sources, and it did not obtain health professionals’ perspectives.^36^ In addition, this study is the first to empirically demonstrate the considerable extent to which providers rely on their own professional judgment when providing euthanasia in Belgium.

The results in this study support international research which demonstrates that providers appreciate the clear boundaries established by AD legislation for euthanasia practice.^37^ They also support research which demonstrates the supportive and clarifying role played by extra-legal sources of regulation,^38^ as well as the influential nature of euthanasia criminal trials on practice.^39^

The results in this study provide some important evidence that nuances claims made in the literature describing limitations of the Act’s retrospective oversight mechanisms. A critique written by Raus, Vanderhaegen, and Sterckx, for instance, identified limitations in the FCECE’s retrospective oversight of performed euthanasia cases.^40^ These included an inability for the FCECE to detect and address failures to report euthanasia cases and to identify falsified, reported information. Participants in this study also identified these limitations and noted it is possible that euthanasia cases are not reported or not reported fulsomely. However, the participants described that they do choose to report and consider this obligation to be important. These findings did not support the contention that current retrospective oversight mechanisms automatically lead to non-compliance and practitioners failing to respect their obligations surrounding oversight. Some participants in this study reported making mistakes in the euthanasia assessment process, such as forgetting to report or to observe the 1-month waiting period required in some cases though these mistakes were neither intentional nor ill-intended.

### D. Implications for policy

These findings have implications for the consistency, quality, and control of Belgian euthanasia practice.

The extent to which health professionals rely on their professional judgment when providing euthanasia has implications for the consistency of euthanasia provision. While some differences in how euthanasia is provided might not necessarily be undesirable, it is problematic where it impairs access or imposes a barrier for eligible patients accessing euthanasia. A reason that healthcare is regulated is to ensure consistent practice.^41^ Relatedly, this potential for inconsistent practice has implications for the quality of care that patients receive. Some institutions, for example, include the ‘palliative filter’ in the euthanasia assessment process, while others do not.^42^ The extent to which the palliative filter contributes to a quality assessment trajectory is not clear. Further research is needed to investigate quality-enhancement measures employed by providers and the extent to which they contribute to the quality of the euthanasia assessment and care processes. This research should also consider the potential implications of such measures for access to euthanasia.

With respect to the control of AD practice, the findings suggest that compliance with the retrospective oversight obligations depends on physicians’ good faith participation. Despite the FCECE not being able to compel compliance, practitioners generally follow the legal requirement to report. In this way, the regulatory framework relies on the professional and deontological integrity of physicians favouring compliance, which appears to induce a high level of compliance. Policymakers may be content with this approach, but if a higher degree of societal control is viewed as desirable, a more elevated form of retrospective oversight could be considered, supported by a more expansive mandate of the FCECE to investigate cases.

The findings in this study are important for policymakers involved in regulating AD internationally. Regulation has an inherently cultural aspect, and it reflects the specific context in which it operates.^43^ That Belgian providers can exercise some degree of clinical discretion, enjoy a high degree of professional autonomy and external non-intervention in decision-making, and are subject only to retrospective, as opposed to prospective, oversight reflects this cultural context. A different approach may be needed in different settings. For instance, evidence from the Victorian voluntary assisted dying system in Australia suggests that practitioners find comfort in and feel confident applying AD legislation that is narrowly framed and not very conducive to a high degree of professional autonomy.^44^ However, models such as the Victorian system may be less flexibly applied by providers. For example, they may make it more difficult for providers to assess patients who are in the ‘grey zone’ of eligibility. Providers in Belgium may more readily be able to provide euthanasia for these patients where they judge that the law intended to grant access to the specific patient.^45^ Future research should compare these features of the Belgian model of AD regulation with approaches adopted in other jurisdictions. This would facilitate the generation of an evidence base for jurisdictions yet to permit AD regarding the characteristics and possible implications of each model of AD regulation.

### E. Strengths and limitations

This study is the first to investigate how euthanasia regulation shapes health professionals’ euthanasia practice in Belgium. The included participants’ cultural and ethnic identity is homogeneous, and there is a possibility that participants with specific views on euthanasia and euthanasia regulation were not reflected in the study. In addition, the focus of the study on regulation and practice in the Dutch-speaking regions of Belgium meant that Walloon euthanasia practice and regulation were not explored. This decision reflected research demonstrating that extra-legal sources of regulation are different between Flanders and Wallonia and that there is evidence of differing practices.^46^ Accordingly, there are some important perspectives that were not included in the study.

In March 2024, after the participants in this study had been interviewed, the Act was amended. These amendments, which entered into force in April 2024, reflect recent case law from the European Court of Human Rights and Belgian Constitutional Court on the subject of Belgian euthanasia practice.^47^ The changes mean that there is no longer a sealed part of the registration form in which the performing physician’s identity is hidden from the FCECE unless they consider there is a need to open it. The registration form is now one continuous document, and the recent amendments also included in the Act specific (and differing) sanctions relating to the breach of one of its ‘basic’ as opposed to ‘procedural’ provisions. It is important that future research considers how these amendments may alter providers’ views of the Act and its retrospective oversight mechanisms.

Not all participants in this study were interviewed in their Dutch mother tongue. The options given to participants, subsequent procedures, and supports implemented to ensure that participants were comfortable participating in the interview in their chosen language were described above. Reflexive reflections about the influence of the language of the interview on the interview data were incorporated into subsequent analysis. Data analysis also reflected reflexive journal entries relating to the positioning of M.A., L.W., and B.P.W. as being culturally and geographically external to the research participants, and the positionings of K.C. and L.D. as being embedded within the participants’ own geographical and cultural setting.

## V. CONCLUSION

This study is the first to investigate the influence of the Belgian euthanasia regulatory landscape (and individual ‘sources’ of regulation) on health professionals’ euthanasia practices. We generated three overarching themes: the euthanasia legislation is a valuable, boundary-setting instrument; it provides limited guidance, leaving space for gap filling and other forms of regulation; and relying on professional judgment can make practitioners feel vulnerable. Important findings include the considerable extent to which euthanasia practice is shaped by providers’ professional judgment, and that practitioners generally comply with the retrospective oversight mechanisms, despite those mechanisms being unable to compel compliance. The Belgian model of euthanasia regulation has implications for the consistency, quality, and control of euthanasia practice. Policymakers in Belgium and internationally should be aware of the advantages and disadvantages associated with this and other regulatory models for AD.

## Supplementary Material

fwaf003_Supplementary_Data

## Footnotes

1 Joachim Cohen and Kenneth Chambaere, ‘Increased Legalisation of Medical Assistance in Dying: Relationship to Palliative Care’ (2022) 13 BMJ Supportive & Palliative Care 178.

2 The Belgian euthanasia legislation has not been officially translated into English. References to the legislation in this article are references to the official English translation of the legislation contained in a Belgian Constitutional Court judgement: *Judgement 153/2015* [2015] Constitutional Court.

3 For a comprehensive discussion of these requirements, see Kasper Raus, ‘The Extension of Belgium’s Euthanasia Law to Include Competent Minors’ (2016) 13 Journal of Bioethical Inquiry 305.

4 Ben P White, Lindy Willmott and Eliana Close, ‘Better Regulation of End-Of-Life Care: A Call for a Holistic Approach’ (2022) 19 Journal of Bioethical Inquiry 683.

5 Eric Byrnes, Alasdair Iain Ross and Mike Murphy, ‘A Systematic Review of Barriers and Facilitators to Implementing Assisted Dying: A Qualitative Evidence Synthesis of Professionals’ Perspectives’ (2022) 90 OMEGA—Journal of Death and Dying 1137.

6 Jamie K Fujioka and others, ‘Implementation of Medical Assistance in Dying: A Scoping Review of Health Care Providers’ Perspectives’ (2018) 55 Journal of Pain and Symptom Management 1564.

7 See, eg, Joke Lemiengre and others, ‘Impact of Written Ethics Policy on Euthanasia From the Perspective of Physicians and Nurses: A Multiple Case Study in Hospitals’ (2010) 1 AJOB Primary Research 49; Joke Lemiengre and others, ‘Content Analysis of Euthanasia Policies of Nursing Homes in Flanders (Belgium)’ (2009) 12 Medicine, Health Care and Philosophy 313; Yanna Van Wesemael and others, ‘Role and Involvement of Life End Information Forum Physicians in Euthanasia and Other End-of-Life Care Decisions in Flanders, Belgium’ (2009) 44 Health Services Research 2180; Yanna Van Wesemael and others, ‘Establishing Specialized Health Services for Professional Consultation in Euthanasia: Experiences in the Netherlands and Belgium’ (2009) 9 BMC Health Services Research 220.

8 White, Willmott and Close (n 4).

9 For a discussion of the Belgian euthanasia law, see, eg, Tinne Smets and others, ‘Attitudes and Experiences of Belgian Physicians Regarding Euthanasia Practice and the Euthanasia Law’ (2011) 41 Journal of Pain and Symptom Management 580. For a discussion of a training programme and consultation service on euthanasia in Belgium, see, eg, Yanna Van Wesemael and others, ‘Implementation of a Service for Physicians’ Consultation and Information in Euthanasia Requests in Belgium’ (2012) 104 Health Policy 272. For a discussion of euthanasia policies, see, eg, Joke Lemiengre and others, ‘How Do Hospitals Deal with Euthanasia Requests in Flanders (Belgium)? A Content Analysis of Policy Documents’ (2008) 71 Patient Education and Counseling 293.

10 White, Willmott and Close (n 4).

11 Madeleine Archer and others, ‘Mapping Sources of Assisted Dying Regulation in Belgium: A Scoping Review of the Literature’ [2023] OMEGA—Journal of Death and Dying (Early online); Madeleine Archer and others, ‘What Domains of Belgian Euthanasia Practice Are Governed and by Which Sources of Regulation: A Scoping Review’ [2023] OMEGA—Journal of Death and Dying 00302228231221839 (Early online).

12 Archer and others, ‘Mapping Sources of Assisted Dying Regulation in Belgium: A Scoping Review of the Literature’ (n 11).

13 Archer and others, ‘What Domains of Belgian Euthanasia Practice Are Governed and by Which Sources of Regulation’ (n 11).

14 See, eg, Kasper Raus, Bert Vanderhaegen and Sigrid Sterckx, ‘Euthanasia in Belgium: Shortcomings of the Law and Its Application and of the Monitoring of Practice’ (2021) 46 Journal of Medicine and Philosophy 80; Herman Nys, ‘A Discussion of the Legal Rules on Euthanasia in Belgium Briefly Compared with the Rules in Luxembourg and the Netherlands’ in David Albert Jones, Chris Gastmans and C MacKellar (eds), *Euthanasia and Assisted Suicide: Lessons From Belgium* (1st edn, Cambridge University Press 2018).

15 Julia Black, ‘Critical Reflections on Regulation’ (2002) 27 Australian Journal of Legal Philosophy 1, 4–8.

16 Joachim Cohen and others, ‘Cultural Differences Affecting Euthanasia Practice in Belgium: One Law but Different Attitudes and Practices in Flanders and Wallonia’ (2012) 75 Social Science & Medicine 845.

17 See, eg, Tinne Smets and others, ‘Reporting of Euthanasia in Medical Practice in Flanders, Belgium: Cross Sectional Analysis of Reported and Unreported Cases’ (2010) 341 BMJ (Clinical research ed) c5174; Tinne Smets and others, ‘Euthanasia in Patients Dying at Home in Belgium: Interview Study on Adherence to Legal Safeguards’ (2010) 60 British Journal of General Practice e163.

18 Black (n 15) 26.

19 Queensland University of Technology, ‘Home’ (*Optimal Regulation of Voluntary Assisted Dying*) <https://research.qut.edu.au/voluntary-assisted-dying-regulation/> accessed 23 April 2024.

20 Joseph Alex Maxwell, *A Realist Approach for Qualitative Research* (SAGE Publications 2012).

21 Els Inghelbrecht and others, ‘The Role of Nurses in Physician-Assisted Deaths in Belgium’ (2010) 182 Canadian Medical Association Journal 905; Nele De Bal and others, ‘Involvement of Nurses in Caring for Patients Requesting Euthanasia in Flanders (Belgium): A Qualitative Study’ (2006) 43 International Journal of Nursing Studies 589.

22 Archer and others, ‘Mapping Sources of Assisted Dying Regulation in Belgium: A Scoping Review of the Literature’ (n 11).

23 Sue L Motulsky, ‘Is Member Checking the Gold Standard of Quality in Qualitative Research?’ (2021) 8 Qualitative Psychology 389; Sarah J Tracy, ‘Qualitative Quality: Eight “Big-Tent” Criteria for Excellent Qualitative Research’ (2010) 16 Qualitative Inquiry 837.

24 Virginia Braun and Victoria Clarke, ‘Using Thematic Analysis in Psychology’ (2006) 3 Qualitative Research in Psychology 77; Virginia Braun and Victoria Clarke, *Thematic Analysis: A Practical Guide* (SAGE Publications 2022).

25 Braun and Clarke, ‘Using Thematic Analysis in Psychology’ (n 24); Virginia Braun and Victoria Clarke, ‘Can I Use TA? Should I Use TA? Should I Not Use TA? Comparing Reflexive Thematic Analysis and Other Pattern‐based Qualitative Analytic Approaches’ (2021) 21 Counselling and Psychotherapy Research 1.

26 Kirsti Malterud, Volkert Dirk Siersma and Ann Dorrit Guassora, ‘Sample Size in Qualitative Interview Studies: Guided by Information Power’ (2016) 26 Qualitative Health Research 1753.

27 Braun and Clarke, ‘Using Thematic Analysis in Psychology’ (n 24); Braun and Clarke, *Thematic Analysis: A Practical Guide* (n 24) 270.

28 Allison Tong, Peter Sainsbury and Jonathan Craig, ‘Consolidated Criteria for Reporting Qualitative Research (COREQ): A 32-Item Checklist for Interviews and Focus Groups’ (2007) 19 International Journal for Quality in Health Care 349.

29 Virginia Braun and Victoria Clarke, ‘Supporting Best Practice in Reflexive Thematic Analysis Reporting in Palliative Medicine: A Review of Published Research and Introduction to the Reflexive Thematic Analysis Reporting Guidelines (RTARG)’ (2024) 38 Palliative Medicine 608.

30 Gill Highet and others, ‘Development and Evaluation of the Supportive and Palliative Care Indicators Tool (SPICT): A Mixed-Methods Study’ (2014) 4 BMJ Supportive & Palliative Care 285.

31 Van Wesemael and others, ‘Establishing Specialized Health Services for Professional Consultation in Euthanasia: Experiences in the Netherlands and Belgium’ (n 7).

32 Madeleine Archer and others, ‘Health Professionals’ Perspectives on the First Belgian Euthanasia Trial: A Qualitative Study’ [Under Review]; Marc De Hert, Sien Loos and Kristof Van Assche, ‘The Belgian Euthanasia Law under Scrutiny of the Highest Courts’ (2023) 24 The Lancet Regional Health—Europe 1; Marc De Hert and others, ‘Media Coverage of Belgium’s First Criminal Case Concerning Euthanasia for Psychiatric Patients: A Content Analysis of Flemish Newspapers and Magazines’ (2023) 13 Frontiers in Psychiatry 1050086; Marc De Hert and others, ‘Improving Control over Euthanasia of Persons with Psychiatric Illness: Lessons from the First Belgian Criminal Court Case Concerning Euthanasia’ (2022) 13 Frontiers in Psychiatry 933748.

33
*Watch Dateline: Allow Me To Die* <https://www.sbs.com.au/ondemand/news-series/dateline/dateline-2015/dateline-s2015-ep31/517459523808> accessed 20 June 2024.

34 Natasia Hamarat and others, ‘Exploring Contemporary Forms of Aid in Dying: An Ethnography of Euthanasia in Belgium and Assisted Suicide in Switzerland’ (2022) 46 Death Studies 1593; Herman Nys and Paul Schotsmans, ‘Professional Autonomy in Belgium’ (2000) 21 Theoretical Medicine and Bioethics 425.

35 Archer and others, ‘Mapping Sources of Assisted Dying Regulation in Belgium: A Scoping Review of the Literature’ (n 11); Archer and others, ‘What Domains of Belgian Euthanasia Practice Are Governed and by Which Sources of Regulation’ (n 11); Monica Verhofstadt and others, ‘Psychiatric Patients Requesting Euthanasia: Guidelines for Sound Clinical and Ethical Decision Making’ (2019) 64 International Journal of Law and Psychiatry 150; Monica Verhofstadt and others, ‘Concrete Experiences and Support Needs Regarding the Euthanasia Practice in Adults With Psychiatric Conditions: A Qualitative Interview Study Among Healthcare Professionals and Volunteers in Belgium’ (2022) 13 Frontiers in Psychiatry 859745; Chris Gastmans, Joke Lemiengre and Bernadette Dierckx de Casterlé, ‘Development and Communication of Written Ethics Policies on Euthanasia in Catholic Hospitals and Nursing Homes in Belgium (Flanders)’ (2006) 63 Patient Education and Counseling 188.

36 Archer and others, ‘Mapping Sources of Assisted Dying Regulation in Belgium: A Scoping Review of the Literature’ (n 11).

37 Harm van Marwijk and others, ‘Impact of Euthanasia on Primary Care Physicians in the Netherlands’ (2007) 21 Palliative Medicine 609; Smets and others (n 9).

38 Byrnes, Ross and Murphy (n 5); Lemiengre and others, ‘Impact of Written Ethics Policy on Euthanasia From the Perspective of Physicians and Nurses’ (n 7); Eliana Close, Jocelyn Downie and Ben P White, ‘Practitioners’ Experiences with 2021 Amendments to Canada’s Medical Assistance in Dying Law: A Qualitative Analysis’ (2023) 17 Palliative Care and Social Practice 26323524231218282.

39 Archer and others, ‘Health Professionals’ Perspectives on the First Belgian Euthanasia Trial: A Qualitative Study’ (n 32); Verhofstadt and others (n 35); Eva Constance Alida Asscher and Suzanne van de Vathorst, ‘First Prosecution of a Dutch Doctor since the Euthanasia Act of 2002: What Does the Verdict Mean?’ (2020) 46 Journal of Medical Ethics 71.

40 Raus, Vanderhaegen and Sterckx (n 14).

41 White, Willmott and Close (n 4).

42 The ‘palliative filter’ refers to the patient seeking access to euthanasia being required to have one or more consultations with a palliative care team or professional before their euthanasia request can be progressed: Lemiengre and others, ‘How Do Hospitals Deal with Euthanasia Requests in Flanders (Belgium)? A Content Analysis of Policy Documents’ (n 9).

43 Colin Scott, ‘Analysing Regulatory Space: Fragmented Resources and Institutional Design’ (2001) [Summer] Public Law 283; Leigh Hancher and Michael Moran, ‘Organizing Regulatory Space’ in Leigh Hancher and Michael Moran (eds), *Capitalism, Culture, and Economic Regulation* (Clarendon Press 1989); Joachim Cohen and others (n 16).

44 Lindy Willmott and others, ‘Participating Doctors’ Perspectives on the Regulation of Voluntary Assisted Dying in Victoria: A Qualitative Study’ (2021) 215 Medical Journal of Australia 125; Marcus Sellars and others, ‘Medical Practitioners’ Views and Experiences of Being Involved in Assisted Dying in Victoria, Australia: A Qualitative Interview Study among Participating Doctors’ (2022) 292 Social Science & Medicine 114568.

45 Ben P White and others, ‘Comparative and Critical Analysis of Key Eligibility Criteria for Voluntary Assisted Dying under Five Legal Frameworks’ (2021) 44 University of New South Wales Law Journal 1663.

46 Cohen and others (n 16).

47 Loi portant dispositions en matière de digitalisation de la justice et dispositions diverses [Law on the digitization of justice and miscellaneous provisions] 2024; De Hert, Loos and Van Assche (n 32).

