# Supplementary material for: How does regulation influence euthanasia practice in Belgium? A qualitative exploration of involved doctors’ and nurses’ perspectives
Source: Med Law Rev. 2025 Jan 29;33(1):fwaf003. doi: 10.1093/medlaw/fwaf003 (PMC11783285; doi:10.1093/medlaw/fwaf003)
Supplement: fwaf003_Supplementary_Data [file fwaf003_supplementary_data.zip › OP-MEDL250003_PECorr_CmtAttachmentsFolder_SupplementaryFile2Nonanonymised170125.docx]

**How does regulation influence euthanasia practice in Belgium? A qualitative exploration of involved doctors’ and nurses’ perspectives**

**Supplementary Material 2**

**Consolidated criteria for reporting qualitative studies (COREQ): 32-item checklist**

| **COREQ item** | **Discussion of this item** | **Section of the article which presents this information** |
| --- | --- | --- |
| Domain 1: Research team and reflexivity | | |
| 1. Interviewer/facilitator | Participants were able to indicate their comfort participating in English, Dutch, or being assisted to participate in English.  MA led the 14 interviews conducted in English. LW co-interviewed in 4 interviews.  MA led the 4 interviews in which the participant was supported to participate in English with the presence of a Dutch-speaking member of the research team. This was KC in 3 interviews and LD in 1 interview.  KC led the 2 interviews undertaken in Dutch. | Method |
| 1. Credentials | MA: BSc-LLB (First Class Hons), GradDipLegPrac | N/A |
| 1. Occupation | MA is a PhD candidate in the Australian Centre for Health Law Research at the Queensland University of Technology | N/A |
| 1. Gender | Female | N/A |
| 1. Experience and training | MA has been researching assisted dying law and practice since 2020 and worked as a research assistant on projects undertaking qualitative research since 2018. MA received training on conducting qualitative research prior to commencing data collection for this study.  The other members of the research team have extensive experience conducting research on assisted dying. Members of the research team were present in several interviews to facilitate the interview. They provided further training to MA subsequent to those interviews. | N/A |
| 1. Relationship established | One of the recruitment methods used in the study was recruitment through the professional networks of the research team (this is described in the Methods section). Where this recruitment method was used, the study participants had a pre-existing relationship with the relevant member of the research team prior to their participating in an interview.  MA did not have any pre-existing relationship with any of the research participants. MA led all the interviews except for the two led by KC. KC did not have any pre-existing relationship with these participants. | N/A |
| 1. Participant knowledge of the interviewer | The informed consent form contained information about the aims and purposes of the study. Participants were required to read and sign this document prior to their participation in the study. This information described that the research was being undertaken as part of MA’s doctoral studies and that it was also part of a broader research investigation.  The aims and purposes of the research were described to participants at the beginning of the interview. Participants were given the opportunity at that stage (and subsequently) to ask questions about the aims of the research and MA’s background and interest in the research. Several participants took the opportunity to do so. | N/A |
| 1. Interviewer characteristics | This study forms part of MA’s doctoral studies. It is also part of a broader project exploring assisted dying regulation in Australia, Canada, and Belgium. | N/A |
| Domain 2: Study design | | |
| 1. Methodological orientation and theory | A critical realist approach to the research was adopted. | Method |
| 1. Sampling | Selection of participants in this study was purposive. Recruitment through advertising the study in English and Dutch through relevant organisations was supplemented in two ways. (1) Recruitment occurred through the professional networks of the research team and (2) snowball approaches to participant recruitment were also used. | Method |
| 1. Method of approach | Participants either contacted a member of the research team via email, or they were directly contacted by a member of the research team via email. | N/A |
| 1. Sample size | There were 20 one-off interviews conducted for this study. | Method |
| 1. Non-participation | No participants withdrew from the study after they had participated. | N/A |
| 1. Setting of data collection | Microsoft Teams videoconferencing software was used for the semi-structured interviews. Participants were informed that they could attend from a location of their choosing, which was generally their workplace or their home. | Method |
| 1. Presentence of non-participants | No non-participants were present in the interviews. Only the researcher or researchers and the participant were present during the interview. | N/A |
| 1. Description of sample | Information about the sample is provided in the Results section including information about participants’ demographic characteristics. | Results |
| 1. Interview guide | An interview guide was used in the study. It was developed for the broader study in which this specific study sits, and was adapted for this study. The interview guide is presented in supplementary file 1 and its content is described in the Method section. | Method; Supplementary Material 1 |
| 1. Repeat interviews | There were no repeat interviews conducted for this study. All interviews were one-off. | N/A |
| 1. Audio/visual recording | The interviews were conducted using a recorded meeting using Microsoft Teams videoconferencing. | Method |
| 1. Field notes | MA made field notes during and after the interviews. Reflexive notes were also made after the interview. Both the field notes and reflexive journal entries were reflected in subsequent data analysis. | Method |
| 1. Duration | Interviews lasted for a median length of 94 minutes (range: 65 minutes to 111 minutes). | Results |
| 1. Data saturation | The concept of ‘information power’ was used to determine the point after which no further interviews were conducted. The research team made this determination based on an assessment of the factors relevant to ‘information power’ such as the study’s aim and analysis strategy. | Method |
| 1. Transcripts returned | All participants were sent their transcript once it had been prepared. Participants could add to their transcript, remove information from it, or change it. They were given a period of two weeks in which to do so (or longer if they requested). Some chose not to alter their transcript.  The purpose of giving participants this opportunity was to ensure that participants could clarify any insights provided in their transcript, or remove insights which they did not want to be published. As many participants participated in English (not their mother tongue) this also gave them the opportunity to clarify any insights that they would have expressed differently, had they been using their mother tongue. | Method |
| Domain 3: Analysis and findings | | |
| 1. Number of data coders | MA coded the data. Final themes were reached through ongoing discussions with, and feedback provided by the research team. | Method |
| 1. Description of the coding tree | A description of the coding tree has not been included. | N/A |
| 1. Derivation of themes | The approach taken to coding in this study was inductive (that is, driven by the data) not deductive (driven by the study’s theoretical framework).  There were no *a priori* codes developed and applied based on regulatory theory in this study. | Method |
| 1. Software | Data analysis was facilitated by NVivo (release 1.6.1). | Method |
| 1. Participant checking | Participants did not give feedback on the themes nor broader findings generated in the study. | N/A |
| 1. Quotations presented | Illustrative participant quotations are included in the Results section. Where this is done, the participant’s unique identification number is provided. Some of the quotes have been altered for language and to preserve the anonymity of the participant or others. Where this has been done, the meaning of the quotation has not been altered. | Results |
| 1. Data and findings consistent | Yes. |  |
| 1. Clarity of major themes | The three major themes are clearly presented in the Results section. | Results |
| 1. Clarity of minor themes | A description of diverse cases and minor themes is present in the Results section. | Results |
