# Supplementary material for: How does regulation influence euthanasia practice in Belgium? A qualitative exploration of involved doctors’ and nurses’ perspectives
Source: Med Law Rev. 2025 Jan 29;33(1):fwaf003. doi: 10.1093/medlaw/fwaf003 (PMC11783285; doi:10.1093/medlaw/fwaf003)
Supplement: fwaf003_Supplementary_Data [file fwaf003_supplementary_data.zip › fwaf003_Supplementary_Data.zip!/227b8_SuplementaryFile3Nonanonymised090125.docx]

**How does regulation influence euthanasia practice in Belgium? A qualitative exploration of involved doctors’ and nurses’ perspectives**

**Supplementary Material 3**

**Reflexive Thematic Analysis Reporting Guidelines (RTARG) – Virginia Braun & Victoria Clarke**^[1]^

This supplementary material is included to provide more information on the consideration given to the Reflexive Thematic Analysis Reporting Guidelines (‘RTARG’) in the conduct and reporting of this study. Braun and Clarke’s advice for each section of a research paper which uses reflexive thematic analysis (‘RTA’) is presented below, with a corresponding discussion of that advice and how it was reflected in the article (where feasible and appropriate).

| **RTARG advice** | **Discussion of this advice and how it is reflected in this article, where feasible and appropriate** |
| --- | --- |
| **The Introduction** | |
| ***Background and rationale*** | |
| Provide a robust context and rationale for the proposed research in the Introduction. | The introduction section of the article discusses existing research, relevant regulatory theory and provides context in relation to this research on the impact of regulation on Belgian euthanasia practice. This section of the article identifies how the new knowledge generated in this article adds to and enriches existing knowledge that exists in relation to 1) individual sources of Belgian euthanasia regulation such as training programs, legislation, and case law and 2) the comprehensive suite of sources of regulation which make up the Belgian euthanasia regulatory landscape. |
| Clearly articulate a research question – one that is methodologically coherent | The introduction section of the article presents a clear statement of the article’s methodologically coherent research question. It is: ‘how does current Belgian euthanasia regulation (including gaps in regulation) influence providers’ euthanasia practice?’ This research question is appropriately answered through the semi-structured interview study described in the methodology section of the article. |
| ***“Owning your own perspectives”*** | |
| Include information on guiding theoretical assumptions and other (e.g. explanatory) theory informing use of TA) | We adopt a critical realist ontological and epistemological positioning in this research. We articulate this in the Methodology section of the article. |
| Report in a way that is consistent with stated theoretical assumptions throughout | We made efforts to report this research in a way that is consistent with theoretical assumptions. |
| Evidence methodological coherence/integrity in both the research and the report | We made efforts to evidence methodological coherence in the research conducted for and reported in this article. |
| Show evidence of reflexive practice | The Methodology section of the article describes researcher reflexivity and the use of a reflexive journal in the coding and theme development processes. That section provides an example of the specific probing that MA undertook in those journal reflections. |
| Write in a methodologically coherent style | We made efforts to write the article in a way that reflects the researchers as active contributors to and influences upon the research. |
| **The Methodology** | |
| ***Participants/data items*** | |
| Describe selection of participants/data items | We describe the selection of participants in the Methodology section. |
| Describe number of participants/data items; provide a rationale or explanation around dataset or participant group size/composition | The number of participants and a rationale around the participant group size and composition is provided in the Methodology section. This study used the non-positivist qualitative concept of ‘information power’ to determine the point at which interviews ceased. |
| Discuss characteristics of participants/data items | The characteristics of the selection of participants included in the study are presented in the Results section of the article. This information is provided in aggregate form in Table 1. While we acknowledge that aggregating this information limits the extent to which each participant’s characteristics can be understood in the context of the other participants, we consider that the information provided appropriately situates the participant group. In addition, this aggregation reflects that the most important consideration with this data was the need to ensure participant anonymity. |
| Detail ethical approval and ethical code/principles followed, participant informed consent, etc | Information on the ethical approval that the study received, and the provision of informed consent is provided in the Methodology section. |
| ***Dataset generation*** | |
| Provide some rationale for method(s) for data generation/data item sources chosen | Semi-structured interviews are a useful method for data generation in this study. Semi-structured interviews allow the researchers to follow a relatively structured approach to the interview (and the use of an interview guide to guide the discussion) while permitting divergences from that structure when the participant shares insights or perspectives that are unique or warrant a deeper exploration. |
| Describe development and/or characteristics of data generation tools | An interview guide was developed and used in this study which guided data generation. This interview guide is described in the Methodology section of the article and is provided in Supplementary Material 1. |
| Include details such as modality and/or setting of data generation, time frame, and other pertinent procedural information | We undertook semi-structured interviews using Microsoft Teams videoconferencing software at the time and location of the participant’s choosing. To reflect the time difference between Australia and Belgium, participants were given the option of participating in the morning or in the evening at a specific time they selected, and which suited them. Interviews were undertaken from September 2022 to March 2024. This is described in the Methodology section of the article. |
| Describe who conducted any interactive data generation (which author or research role), and how | The roles that each researcher had in the data generation process is described in the Methodology section. |
| Describe the size/scope of the dataset and dataset items | The range and median length of the interviews are described in the Results section. |
| Describe, and if relevant explain, any preparation of data for analysis | The use of transcription and translation of the interviews undertaken in Dutch is described in the Methodology section. The use and function of obtaining further reflections from participants on their transcript is also described in that section. |
| ***Data analysis*** | |
| Provide some rationale for the use of RTA, and were relevant, for combining RTA with other approaches and procedures | The rationale for using RTA is described in the Methodology section. |
| Describe specific orientation to RTA | The inductive orientation to reflective thematic analysis is described in the Methodology section. |
| Discuss how the researcher(s) engaged with the analytic process | MA analysed the data, with guidance from the other authors. MA followed the six phases of Braun and Clarke’s reflexive thematic analysis, consisting of: data familiarisation, coding, generating initial themes, developing and reviewing themes, refining, defining and naming themes, and writing up.^[2]^ Themes were settled as a result of discussions within the research team. |
| Where more than one person is involved, describe who analysed the data (author or research role) | MA primarily analysed the data. Several discussions took place with the research team to discuss the framing, content, and relationship between themes. Decisions taken reflecting each of these characteristics reflected this discussion within the research team. Inter-coder agreement measures and consensus coding approaches were not used as they were not consistent with the theoretical assumptions underlying this research. |
| Use language to describe the process and products of RTA that is coherent with the values and assumptions of RTA | The language used in the Methodology section is coherent with the values and assumptions of RTA. For example, the methodology section of the article describes how themes were ‘generated’ or ‘constructed’ by the researchers and through the analytical process rather than ‘discovered’. Though Braun and Clarke prefer the term ‘Analysis’ to ‘Results’ (to describe the section in which the themes are presented), the term ‘Results’ is used in this study as this term is likely to be the most familiar for (and avoid confusion for) readers. We considered that naming the ‘results’ section ‘analysis’ may have lead readers to view that section as further elaboration of the methodology rather than a discussion of the outputs of the thematic analysis process. |
| **The Analysis** | |
| ***Reporting the data analysis*** | |
| Provide an overview of themes or thematic structure | A visual overview of themes and their thematic structure is included in the Results section of the article. This structure articulates the overarching themes, themes and sub themes and the relationships between them. It is consistent with the narrative about the themes that follows it. |
| Ensure theme conceptualisation is appropriate to RTA, and any divergences are justified and explained | Themes generated in this study reflect patterns of shared meaning, rather than topic summaries. Some of the themes generated in the analysis are sematic and some are latent. |
| Name themes appropriately | The theme names reflect the essence of each theme. |
| Report themes in sufficient detail and depth | The themes are reported in considerable detail and depth; data extracts (illustrative participant quotations) are included; and the reporting of themes references both participant data and the researchers’ narrative in relation to that data. |
| Use subtheme judiciously | The themes are multifaceted and complex. Themes and sub-themes were generated during data analysis (as falling under the overarching themes). These ‘levels’ of themes were developed judiciously and reflect the data. |
| Ensure analytic narrative explains the meaning and significance of the data | The analytic narrative explains participant data (encompassing an integration of participants’ data and the researchers’ analytical narrative regarding that data). Descriptions are provided for how illustrative participant quotations (data extracts) provide evidence for the statements made in the reporting of themes. |
| Provide an appropriate balance of analytic narrative and data extracts – both data extracts and analytic narrative matter | The reporting of the themes integrates participants’ data and the researchers’ analytical narrative relating to that data. Participant quotations are provided to illustrate the themes. |
| Demonstrate coherence between analytic narrative and illustrative/evidentiary abstracts | The analytic narrative and data extracts (participant quotations) are coherent. |
| Integrate existing research and theory into the analytic narrative | Existing research and theory are not integrated in the analytic narrative (presented in the Results section of the article). The Discussion section is the section in which the analysis in the study is integrated with existing research and theory. We recognise that Braun and Clarke recommend that this integration occurs. However, this decision was taken to reflect and reinforce the inductive (not deductive) orientation to theme development used in the study and to avoid confusion that a deductive approach to analysis was taken. |
| **The Final Section – A General Discussion or “Conclusions”** | |
| ***Quality, evaluation and conclusions*** | |
| Draw analytic conclusions across themes | The Discussion section of the article discusses the overall implications of the research and describes the narrative that integrates each of the themes. Outcomes for policy are suggested in this section. |
| Discuss implications or directions for future research | Suggestions for future research are included in the Discussion section. Implications of the research for policy are also included in the Discussion section. |
| Use and report quality practices coherent with RTA | The article, including the Discussion section, describes the use of quality practices coherent with a reflexive approach to thematic analysis. For example, the Discussion section reports on the use of reflexive journaling. |
| Evaluate the research from a Big Q standpoint | The Discussion section of the article evaluates this study from a Big Q standpoint. |
| Include reflections on research process and practices, including researcher reflexivity | The use of reflexive research practice is described and reflected upon in the Discussion section of the article. |

1. Virginia Braun and Victoria Clarke, ‘Supporting Best Practice in Reflexive Thematic Analysis Reporting in Palliative Medicine: A Review of Published Research and Introduction to the Reflexive Thematic Analysis Reporting Guidelines (RTARG)’ (2024) 38 Palliative Medicine 608.
2. Virginia Braun and Victoria Clarke, *Thematic Analysis: A Practical Guide* (SAGE Publications 2022).
